# Decoding individual differences in STEM learning from functional MRI data

**DOI:** 10.1038/s41467-019-10053-y

**Published:** 2019-05-02

**Authors:** Joshua S. Cetron, Andrew C. Connolly, Solomon G. Diamond, Vicki V. May, James V. Haxby, David J. M. Kraemer

**Affiliations:** 1 Department of Education, Dartmouth College, Hanover, New Hampshire 03755 USA; 2 Department of Psychology, Harvard University, Cambridge, MA 02138 USA; 3 Department of Psychological and Brain Sciences, Dartmouth College, Hanover, New Hampshire 03755 USA; 4 Thayer School of Engineering, Dartmouth College, Hanover, New Hampshire 03755 USA

**Keywords:** Problem solving, Neural decoding, Human behaviour, Education

## Abstract

Traditional tests of concept knowledge generate scores to assess how well a learner understands a concept. Here, we investigated whether patterns of brain activity collected during a concept knowledge task could be used to compute a neural ‘score’ to complement traditional scores of an individual’s conceptual understanding. Using a novel data-driven multivariate neuroimaging approach—informational network analysis—we successfully derived a neural score from patterns of activity across the brain that predicted individual differences in multiple concept knowledge tasks in the physics and engineering domain. These tasks include an fMRI paradigm, as well as two other previously validated concept inventories. The informational network score outperformed alternative neural scores computed using data-driven neuroimaging methods, including multivariate representational similarity analysis. This technique could be applied to quantify concept knowledge in a wide range of domains, including classroom-based education research, machine learning, and other areas of cognitive science.

## Abstract (web summary)

People differ in their current levels of understanding of many complex concepts. Here, the authors show using fMRI that brain activity during a task that requires concept knowledge can be used to compute a ‘neural score’ of the participant’s understanding.

## Introduction

A principal goal of cognitive science is to be able to characterize how well an individual or intelligent system understands a concept as a result of learning. Traditionally, an individual’s degree of concept knowledge is evaluated using domain-specific knowledge tests: tasks such as pencil-and-paper exams that produce a performance score used as an indicator for conceptual understanding. But acquiring new knowledge also impacts how individuals engage in the world beyond the classroom. Science and engineering students, for example, learn to categorize items in the world in new ways, often along conceptual dimensions that novices cannot perceive. When an individual acquires such knowledge, these new category-based item relationships should theoretically be represented somewhere in the brain. The presence of that representation, if it can be assessed, would in turn constitute a logical neural proxy for conceptual understanding. In the present study, we ask whether it is possible to use an individual’s concept-related brain activity to derive a “neural score” that predicts concept knowledge in a way that converges with performance on a traditional knowledge test. The ability to evaluate concept knowledge using neural data may present new and unique insights into individual differences in learning.

There are many existing ways of assessing an individual’s level of concept knowledge (multiple-choice tests, written exams, oral exams, essay tests, etc.). Typically, the result of these metrics is a single summary statistic (e.g., test score) averaging over a series of concept-related items (e.g., test questions) to indicate an individual’s overall level of understanding of the tested concepts. A more-sensitive alternative to this single test score approach is provided by graph theory: rather than average across concept-related items to produce a scalar indicator, items within a particular conceptual domain can be modeled using a high-dimensional matrix. The values in such a matrix reflect the conceptual distance between each item, such that the matrix as a whole becomes a high-dimensional representation of the concept space. Representational similarity analysis (RSA)^1^ uses these high-dimensional models, known as dissimilarity matrices (DMs), to measure informational content independent of data format. An advantage of RSA is the ability to evaluate the presence or absence of concept-related information within a given system (e.g., nonhuman animals, computer databases, brain activity) using an information processing approach.

In neuroscience research, RSA as well as other multivariate pattern analysis (MVPA) methodologies (e.g., pattern classification) have enabled us to observe that concept-related information is represented in patterns of brain activity^2–17^. These MVPA approaches have often been used to evaluate group differences in representations of previously learned category knowledge, such as categories of objects^5,7,9,14,17^, animals^1,3,4^, numerical magnitude^6,10^, and familiar faces and places^2,7,8,12,15,16^. However, to our knowledge, only one study has used MVPA to evaluate group differences in newly acquired concept representations^11^, and none have used RSA and MVPA approaches to predict individual differences in newly learned concept representations.

The few studies that have explored the relationship between brain activity and individual differences in concept knowledge employed univariate measures and hypothesis-driven approaches. Specifically, these studies tested for correlations between concept-related test performance and brain activity in particular regions of interest, which were often selected a priori^18–21^. Furthermore, these studies relied on metrics derived at the group level or from literature-driven hypotheses about unique brain regions. These approaches are useful for testing hypotheses motivated by the existing literature, and when the relevant information in brain activity can be tested as a change in mean activity level or variance of a given brain region. However, what is lacking in the current literature on the neural predictors of concept knowledge is a bottom–up multivariate approach, useful for when experimenters wish to query the neural data for specific content knowledge on the level of the individual participant. Such an approach would be applicable even in cases where prior knowledge about relevant brain regions is unknown or inconclusive, because it would treat an individual’s brain as a “black box” (i.e., agnostic as to anatomical localization) in the process of evaluating neural activity for representational structure related to concept knowledge.

In the present study, we develop a novel analysis incorporating univariate and multivariate methodologies to derive an individual-level neural score to predict individual differences in concept knowledge. Our aim in developing this method, which we refer to here as an informational network analysis, is to generate a data-driven neural score assessing concept knowledge for an individual, derived by treating the individual’s brain as a black box. This analytical approach is designed to apply generally to any paradigm in which conceptual knowledge can be assessed via a meaningful dimension of similarity among items (e.g., knowledge of mechanical structure categories) independently from other dimensions of similarity (e.g., general visual appearance). We validate our neural score method by comparing individuals’ neural score results to their performance on several convergent tests of concept knowledge. Importantly, in order to meet our criteria this score must be derived using no group-level analyses and without relying upon any prior hypotheses about neural localization. We achieve these aims by using multivariate analytical methods which leverage the richness of the informational content available in brain activity patterns. In addition, as it is unknown whether current methods, such as whole-brain searchlight-based RSA^1^ are sufficient to accomplish this goal on an individual subject level, we compute two alternative neural score variants: a searchlight RSA neural score, and a univariate contrast neural score as a secondary comparison. The informational network analysis successfully outperforms both alternative neural scores and significantly predicts individual differences in STEM concept knowledge, as measured by a functional magnetic resonance imaging (fMRI) task and performance on standardized concept inventories. This method has a wide variety of potential uses for evaluating concept knowledge within informational systems in the domains of cognitive science, education, and machine learning.

## Results

### Free body diagram task performance

**Fig. 1:**
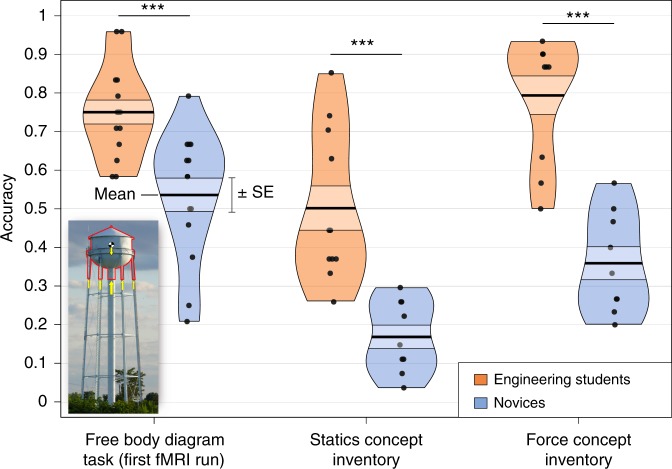
Outcome measures: accuracy scores by group. Group performance on each outcome measure: FBD task performance at the first fMRI run, SCI accuracy score, and FCI accuracy score (between-group *t* test significance values: * = *p* < 0.05, ** = *p* < 0.01, *** = *p* *<* 0.001). Inset: example stimulus from the FBD task. (For a larger version of this example stimulus, see Supplementary Fig. 1)

First we evaluated performance on the free body diagram (FBD) task, the novel concept knowledge test used in this experiment. In this task, participants were presented with photographs of real-world structures and asked to consider the Newtonian forces that must be acting on the indicated section of the structure in each image (see Methods section for full task description). Participants responded via button press to indicate whether they judged a subsequently presented diagram of these forces (Fig. 1, inset; Supplementary Fig. 1) to be labeled correctly or incorrectly. We calculated the average accuracy for the two alternative forced-choice response (chance = 50% accuracy). A two-sample *t* test revealed that on average, engineering students outperformed novices on the FBD task (*M*_Eng._ = 76.4%, *M*_Nov._ = 66.7%, *t*(24.48) = 2.11, *p* = 0.045). But this overall group difference was entirely driven by the group difference in FBD task performance at the first fMRI run (Run 1: *M*_Eng._ = 75.0%, *M*_Nov._ = 53.6%, *t*(23.53) = 3.92, *p* = 0.0007). As the fMRI runs progressed, novices improved on the FBD task despite never receiving feedback on their performance (see Supplementary Fig. 2). As a result, the group differences in FBD task performance disappeared in the subsequent fMRI runs (Run 2: *M*_Eng._ = 72.9%, *M*_Nov._ = 63.4%, *t*(23.24) = 1.82, *p* = 0.08; Run 3: *M*_Eng._ = 76.5%, *M*_Nov._ = 73.5%, *t*(23.06) = 0.51, *p* = 0.6; Run 4: *M*_Eng._ = 81.3%, *M*_Nov._ = 76.5%, *t*(21.08) = 0.99, *p* = 0.3).

The strong group difference effect observed in the first fMRI run of the FBD task makes it the optimal timepoint from which to compute a neural score. A successful neural score collected from the first fMRI run can safely be interpreted to reflect prior mechanical engineering knowledge, independent of experiment-specific mastery of the FBD task. Furthermore, the ceiling effect observed in the later fMRI runs of the FBD task as participants improved on the task during the experiment would complicate the interpretation of any neural scores computed from those later runs, because of the confound between task-specific learning effects and baseline differences in concept knowledge. Therefore, as we proceed with our neural score analysis, we will consider only the first fMRI run.

### Standardized concept knowledge tests

In addition to the FBD task, participants completed two standardized, multiple-choice concept knowledge tests designed to measure mechanical engineering and physics knowledge: the Statics Concept Inventory (SCI)^22^ and the Force Concept Inventory (FCI). These measures were collected in a separate experimental session prior to the fMRI scan session. As expected, these measures differentiated the groups significantly (SCI: *M*_Eng._ = 50.2%, *M*_Nov._ = 16.9%, *t*(14.86) = 5.0, *p* = 0.0002; FCI: *M*_Eng._ = 79.3%, *M*_Nov._ = 35.9%, *t*(16.83) = 6.5, *p* = 5.8 × 10^–6^). These two concept knowledge tests, in conjunction with our FBD task results from the first fMRI run (Fig. 1), constitute a multivariate outcome measure, which we can use to validate our neural score in a linear mixed-effects model. Combining these three concept knowledge tasks into a single multivariate measure is further justified by the strong cross-correlations between each of the task types (FBD vs. SCI: *r* = 0.54; FBD vs. FCI: *r* = 0.55; SCI vs. FCI: *r* = 0.80).

### Neural score results

**Fig. 2:**
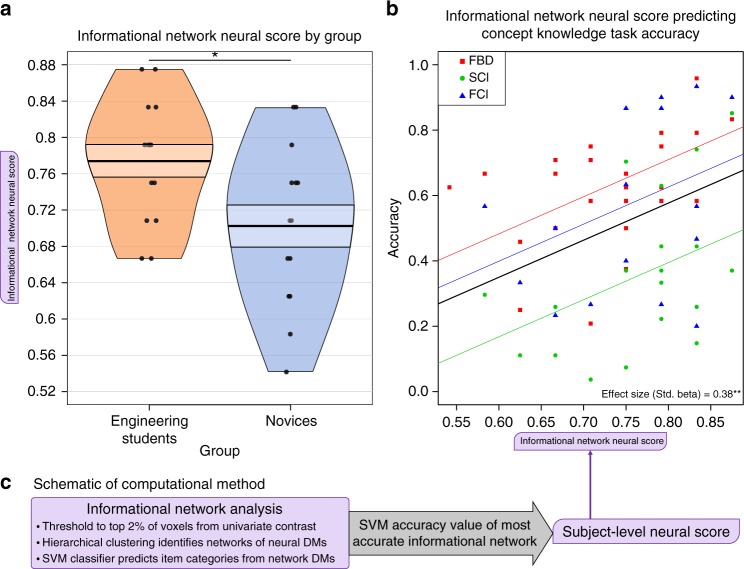
Informational network neural score results. **a** Informational network neural score from the first fMRI run significantly differentiated the participants by group. **b** Informational network neural score significantly predicted concept knowledge in a linear mixed-effects model. The overall regression line is plotted as the thick black line over the observed accuracy data for each outcome measure type (FBD, SCI, and FCI accuracy). Random intercepts for each outcome measure are plotted as the colored lines. Effect size (standardized beta parameter) for the regression model are printed in the lower right corner of the plot. **c** Schematic outlining the computational method for the informational network analysis, used to compute the informational network neural score for each participant. (For between-group *t* test significance value at left and mixed-effects regression coefficient significance value at right: * = *p* < 0.05, ** = *p* < 0.01, *** = *p* *<* 0.001)

**Fig. 3:**
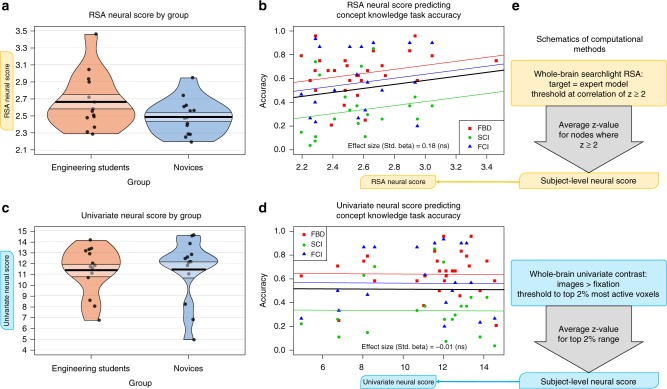
RSA and univariate neural score results. Neither the RSA neural score **a**–**b** nor the univariate neural score **c**–**d** from the first fMRI run significantly differentiated the participants by group, and neither was significantly predictive of concept knowledge. Regression outputs in **b** and **d** follow the same format as Fig. 2b, with effect size (standardized beta parameter) printed at bottom of each plot. Schematics on the right **e** outline the computational method for the RSA and univariate neural scores, following the format used in Fig. 2c

In this section, we will review the results from our primary neural score measure, derived using our novel informational network analysis, as well as the two comparison neural scores, derived using RSA and a univariate contrast (images > baseline), respectively (Figs. 2, 3). Functional data used in all neural score computations included beta parameter estimates for each individual stimulus item, estimating changes in neural activity associated with the stimulus evaluation period of the FBD task (prior to the response period; see Methods section for details).

An overview of the neural score methods discussed here is provided in Table 1. (To review additional neural score variants we computed, see Supplementary Table 1.) To validate our neural score measures, we computed linear mixed-effects models for each neural score predicting concept knowledge (as assessed by FBD, SCI, and FCI accuracy scores). Recall that based on our FBD task ceiling effect, we only considered neural score results from the first fMRI run. The regression output for each neural score model is reported in Table 2.

**Table. 1:** Qualitative differences between neural score methods

Neural score	Neural score description
Informational network	- Univariate voxel threshold - Multivariate score computation - Categorical a priori representational model
Searchlight RSA	- Multivariate voxel threshold - Multivariate score computation - High-dimensional a priori representational model
Whole-brain univariate	- Univariate voxel threshold - Univariate score computation - No a priori representational model

Each neural score derivation involved voxel selection and score computation steps. The informational network score involved a combination of univariate voxel thresholding with multivariate score computation, whereas the other two scores were derived using either entirely univariate or entirely multivariate methods. In addition, the informational network and RSA neural scores incorporated a priori models, whereas the univariate neural score did not. The informational network score incorporated a dimensionality-reduced version of the expert mechanical similarity model by using a category-based SVM classifier, whereas the RSA neural score incorporated the full 24-dimensional expert mechanical similarity model. No neural score method involved any factors with a direct relationship to the concept knowledge measures they were designed to predict

**Table. 2:** Regression results for neural scores predicting concept knowledge

Neural score	*β* parameter estimate	Effect size (*β*_std._)	*p*
Informational network	1.135	0.38	0.007
Searchlight RSA	0.169	0.18	0.2
Whole-brain univariate	−0.001	−0.01	0.9

Regression parameters, effect sizes (standardized betas) and *p* values for each linear mixed-effect model predicting concept knowledge from neural score. Each model includes random intercepts for each participant and each type of concept knowledge test (i.e., FBD task, SCI, and FCI)

### Informational network neural score computation overview

A full description of the informational network analysis can be found in the Methods section. In short, the informational network analysis proceeded as follows: a univariate contrast (stimulus images > fixation baseline) to determine a threshold for voxel inclusion; a whole-brain neural searchlight to identify high-dimensional neural representations of the stimulus set; a bootstrapped hierarchical clustering analysis to reduce the dimensionality of the data; and a support vector machine (SVM) category classifier to identify the degree to which each network represents expert-level category information among the stimuli. The final value used as the informational network score is the SVM categorization accuracy percentage for the most expert informational network (i.e., the network with the highest SVM accuracy).

Importantly, this method does not require as input a predetermined, arbitrary number of networks during the hierarchical clustering step. Instead, a bootstrapping procedure is employed to determine the number of distinct, statistically reliable informational networks present for each individual^23^. Thus, the informational network analysis treats the brain as a black box, drawing all inputs from the internal structure of the neural data. Only at the final step is an external measure of any kind introduced, when a model of conceptual category information is used by a categorical SVM classifier to evaluate each informational network and determine which one contains the most expert-like representational structure.

In order to evaluate the efficacy of the informational network analysis relative to simpler univariate and multivariate methods, we computed two alternative neural scores: a univariate score derived entirely from the whole-brain images > baseline contrast, and a searchlight RSA score derived from a standard whole-brain RSA targeting the full 24-dimensional expert model of mechanical similarity. The RSA neural score was designed as a competitive alternative method to our informational network analysis. The univariate neural score was designed to directly test the contribution of the voxel selection step to the predictive value of the informational network neural score. Derivation steps for each of these alternative neural scores is described in detail in the Methods section. Table 1 shows the qualitative differences between each neural score variant, whereas Table 2 shows the predictive strength of each score for concept knowledge evaluated using linear regression models. Table 3 directly tests each of the neural score models against each other using stepwise model comparisons. Additional alternative neural score models were tested, and we selected the best-performing univariate and RSA methods for comparison. (Results from the alternative neural score configurations not discussed here are reported in Supplementary Table 1.)

**Table. 3:** Direct model comparisons between neural scores

Base model	Composite model	Improvement of composite model over base model (χ^2^)	*p*
Informational network	Informational network + RSA	1.28	0.26
Informational network	Informational network + univariate	0.003	0.96
Searchlight RSA	Informational network + RSA	7.29	0.0069
Whole-brain univariate	Informational network + univariate	7.59	0.0059

Each alternative neural score was directly compared with the informational network neural using stepwise linear mixed model comparisons (likelihood ratio test). The predictive strength of the informational network neural score model was not improved by the addition of either the RSA or the univariate neural score as a second predictor. By contrast, both the RSA and univariate base models were significantly improved by the addition of the informational network model as a second predictor

### Searchlight RSA neural score computation overview

The first alternative neural score method we compared with the informational network score was a multivariate score derived from a whole-brain searchlight RSA^1^. As in the second step of the informational network score, a whole-brain searchlight analysis was run to identify neural representations for the 24 stimulus items. The resultant DMs were each correlated (Spearman’s rho) with a full 24-dimensional expert model of mechanical similarity. After a Fisher *r*-to-*z*-transformation, a threshold of *z* *≥* 2 was applied for voxel selection. The RSA score was finally derived by averaging the *z* values for all the surviving voxels. (A full description of the RSA neural score method can be found in the Methods section.)

To examine whether the voxel threshold applied in this RSA neural score was constraining the predictive value of the RSA neural score, we computed an alternative RSA score, which involved a hierarchical clustering step identical to that used in the informational network analysis. Results from that and other alternative neural score methods not used in these analyses are reported in Supplementary Table 1. These alternative models performed worse than the models included here, therefore we only retained the best-performing RSA and univariate models for comparison to the informational network model.

### Whole-brain univariate neural score computation overview

The second alternative neural score method we compared with the informational network score was a univariate score derived from a whole-brain general linear model (GLM). This is the same univariate GLM used as a voxel mask in the first step of the informational network analysis. The purpose of this univariate score was to directly test the contribution of the voxel selection step to the predictive value of the informational network neural score. Thus, the univariate neural score was derived by simply averaging the *z* values from the most active 2% of voxels for the contrast. (A full description of the univariate neural score method can be found in the Methods section.)

### Predicting concept knowledge from neural scores

Each neural score was modeled separately as a predictor of concept knowledge using linear mixed-effects regression models, with the multivariate dependent measure of FBD task, SCI, and FCI accuracy scores, and random intercepts for participant ID and dependent measure score type (see Methods section for complete details). The neural score generated using our novel Informational network analysis for the first fMRI run significantly predicted concept knowledge using this regression modeling approach (*β*_std._ *=* 0.38, *p* = 0.007; Fig. 2; Table 2). This was the only neural score model we derived that significantly predicted concept knowledge. The neural score derived from a standard RSA approach was not significantly predictive of concept knowledge (*β*_std._ = 0.18, *p* = 0.2; Fig. 3; Table 2), nor was the neural score derived from the univariate contrast of images > fixation baseline (*β*_std._ = −0.01, *p* = 0.9; Fig. 3; Table 2).

Furthermore, the informational network neural score differed significantly by group (*M*_Eng._ = 0.774, *M*_Nov._ = 0.702, *t*(24.41) = 2.37, *p* = 0.03), which is a required feature of any neural score purporting to measure concept knowledge. In other words, any neural score that successfully measures concept knowledge must differentiate engineering students from novices during the same timepoints that the concept knowledge task scores also differentiate these groups; i.e., at the beginning of the fMRI experiment. This group difference in neural score is not observed for the RSA neural score (*M*_Eng._ = 2.67, *M*_Nov._ = 2.49%, *t*(22.12) = 1.73, *p* = 0.1) or for the univariate neural score (*M*_Eng._ = 11.36, *M*_Nov._ = 11.42, *t*(24.4) = −0.07, *p* = 0.9).

### Neural score model comparisons

In order to directly compare each type of neural score, compared each pairwise combination of neural score variants with the base models containing only one neural score predictor. We used likelihood ratio tests to determine the degree of improvement in prediction strength rendered by combining one neural score model with another. The results of these model comparisons are shown in Table 3. In summary, models containing the informational network score as a factor were significantly predictive of the concept knowledge measure, and the model using only the informational network method was not significantly improved by linear combination with any other method.

### Neural score localization

**Fig. 4:**
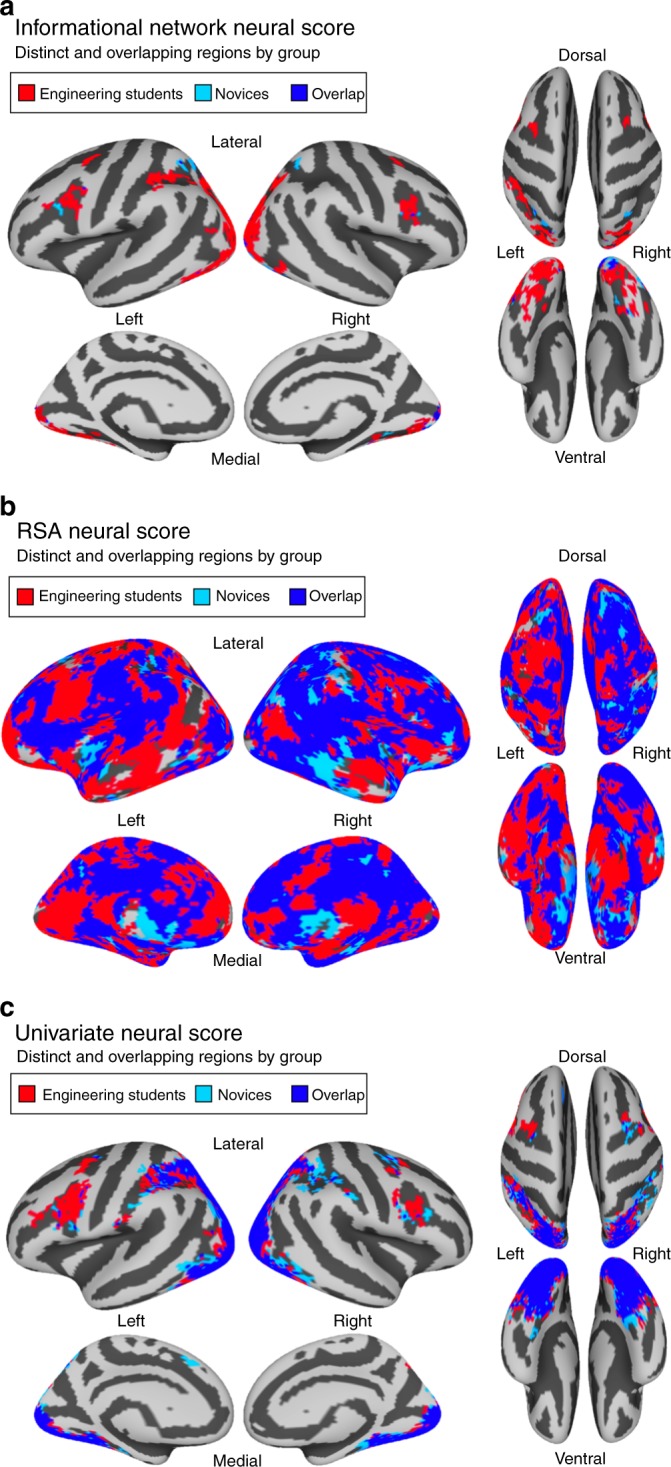
Neural score localizations by group. Regions contributing to neural scores in engineering students are shown in red. Regions contributing to neural scores in novices are shown in light blue. Regions contributing to neural scores in both engineering students and novices are shown in purple. **a**, **b**, and **c** show the informational network, RSA, and univariate neural score maps, respectively

Our aim was to develop a data-driven, individual-level score that predicts performance on a concept knowledge task. Accordingly, we conducted these analyses agnostic to the specific localization in the brain of the data contributing to a given neural score. However, a useful application of the informational network analysis is the ability to project results back onto the brain surface to review the regions where the neural score data are represented at the group level. Accordingly, we report in Fig. 4 the group-specific localizations for the regions contributing to the informational network, RSA, and univariate neural scores data. For the informational network score in particular, these regions correspond to, for each group, the set of cortical areas in which patterns of activity contain information distinguishing the mechanical categories within the stimulus set, thereby reflecting engineering knowledge. In short, we found that the better performance of engineering students on the concept knowledge tasks corresponded to a broader localization of informational network scores compared with novices. The relevant regions for engineering students included both a dorsal stream frontoparietal network, which has been previously implicated in spatial cognition^11,18,25^, and a ventral stream occipito-temporal network previously implicated in visual object identification and categorization^3,4^. For a more-detailed exploration of these data with respect to the localization of different representational geometries corresponding to learned concept knowledge, see Cetron et al. (preprint)^24^.

Figure 4 shows the distinct and overlapping regions contributing to each type of neural score by group. Informational network neural scores showed the most distinct group differences in localization (Fig. 4a), with increasingly overlapping regions contributing to the RSA and univariate neural scores respectively (Fig. 4b, c). (For histograms of the total number of participants with neural scores observed at each region, see Supplementary Fig. 3).

## Discussion

In the present study, we have shown that concept knowledge can be assessed using a neural score derived entirely from an individual’s brain activity patterns using our informational network analysis. The informational network analysis produced neural scores that significantly predicted individual differences in performance on a STEM-domain concept knowledge task (Table 2, Fig. 2). Furthermore, the informational network score method significantly outperformed two alternative data-driven neural scores for predicting concept knowledge (Table 3), which were based on standard neuroimaging methods: whole-brain univariate GLM and whole-brain searchlight RSA (Fig. 3). Finally, we demonstrated that these neural scores can be averaged to produce meaningful group-level maps that reflect localization of conceptual knowledge across subjects (Fig. 4).

It is worth noting that the neural score and the behavioral data it predicts are fully independent from each other. The informational network score is a measure of the presence of expert category-level information in patterns of neural activity. The categories themselves are never mentioned in the course of the experiment, and knowledge of the categories is neither necessary nor sufficient to complete the behavioral task. Furthermore, while the engineering students may be aware that such categories exist, the novices do not have any explicit category-level knowledge about these stimuli. Importantly, the informational network score is equally predictive of performance on the concept knowledge task for both groups.

This finding is additionally remarkable because it is not necessarily the case that any brain region need represent the stimulus set in a way that reflects the expert categories at all, particularly because participants (especially novices) do not consciously know that those categories even exist. Moreover, the behavioral task requires participants to consider only one stimulus at a time, and never asks them to consciously employ a categorical strategy. And yet, not only do we find that there are networks of brain regions networks whose item-level representational structure can be grouped into meaningful informational categories, but we also find that the degree to which that happens in an individual’s brain is predictive of that individual’s demonstrated competence on a related concept knowledge task.

Previous attempts to assess concept knowledge using brain activity have aimed to characterize the neural instantiation of concept knowledge in a way that is common across individuals^3,4,6–8,10,14,17^. The few studies that looked at individual differences in learned concept knowledge relied on previous literature and/or group-level analyses to identify relevant brain regions of interest^18–21^. Our goal in the present study was importantly different: we sought an individualized, data-driven score for predicting learned concept knowledge from brain activity patterns. The existing methods available to analyze concept-related neural data, such as univariate activity contrasts and RSA, were not designed to optimally predict individual differences in concept knowledge, as evidenced by the poor performance of the neural scores derived using these methods. As a result, we sought to create a new analytical tool to achieve that goal. This new informational network analysis accounts for individual variance in univariate brain activity levels, and then employs MVPA to identify concept-related structure represented in brain activity patterns. This multi-stage, data-driven approach allowed for a successful mapping between neural data and a behavioral test of concept knowledge at the individual subject level. Moreover, when these individual scores were averaged together, they produced meaningful group-level results. Those results are consistent with prior work, demonstrating that conceptual knowledge about objects (including visual categorization) includes regions in both ventral occipito-temporal cortex^3,4^ and dorsal regions involved in action representations^11,18,25^. For a more detailed exploration of the neural instantiation of concept knowledge as a function of prior knowledge and experience, see ^24^.

One important feature of the informational network analysis appears to be the dimensionality of the model of concept knowledge it incorporates. An exclusively univariate approach to neural scoring appears to be too simplistic to account for the multidimensional informational content present in patterns of neural activity, as demonstrated by the comparison between the informational network score and the univariate score reported in Table 2. On the other extreme, a standard RSA approach to neural scoring that uses a high-dimensional concept knowledge model works reasonably well, but risks overfitting neural activity to an expert-level a priori model of the concept space that is too specific for the task at hand, which relies predominantly on information that is more closely aligned to the specificity present in category classifications. This discrepancy between the high dimensionality of the RSA model and the reduced dimensionality required to complete the concept knowledge task may help explain why the RSA score did not significantly improve upon the univariate score in predicting task performance. In order to better match the dimensionality of the concept knowledge task at hand, the informational network score incorporated two stages of dimensionality-reduction within the MVPA portion of the computation: hierarchical clustering to reduce redundancy in the neural signal, and SVM classification to identify category-related structure. This category-level model appears to be robust enough to identify relevant patterns of neural activity for assessing concept knowledge, while excluding the item-level idiosyncrasies of our particular expert model of concept knowledge.

A notable limitation of the present study is that the traditional assessment of concept knowledge used here to corroborate our neural score was limited to a single measure: the FBD task completed during the fMRI scan. In future studies, neural scoring can be used to predict more varied measures of concept knowledge, such as classroom-based test scores or long-term concept recall. Additional fine tuning of the methodological approach may also be beneficial and could potentially assess changes in learning over time, even during the scan session. Indeed, other MVPA approaches have successfully incorporated timecourse information^26,27^, and could be utilized in conjunction with the approach we describe here.

Future research may also consider using the neural scoring approach as a predictor of concept knowledge in other conceptual domains beyond physics and engineering, as well as in other areas of research including human developmental learning and even nonhuman animal research. Indeed, the present approach is applicable to any content area in which stimuli can be categorized in a meaningful way that is evident to an expert, and that is not immediately obvious to a novice upon visual presentation of the stimuli. Finally, our informational network analysis can be applied as an information-processing technique to assess concept knowledge within intelligent computational systems in addition to human learners. In this way, we believe this analytical approach can contribute to future work in machine learning domains and in broader areas of cognitive science.

## Methods

### Participants

Thirty-three Dartmouth College students participated in this study. Five participants were dropped from analyses: three had incomplete participation data; two had no above-threshold results from the RSA (described in detail below). The final participant sample consisted of *N* = 28 (*N*_female_ = 16; *M*_age_ = 20.71 years,* SD* = 1.76). Half of the participants (*n* = 14) had no college-level engineering or physics experience (referred to here as “novices”). Half (*n* = 14) were students in the undergraduate engineering major, who, at the time of their participation, had completed or were nearly finished with an intermediate-level solid mechanics engineering course intended for majors which including a laboratory section (referred to here as “engineering students”). All engineering students had previously taken at least one laboratory-based course in advanced physics as a prerequisite for their major. Five of the engineering students had taken additional advanced structural engineering courses as well. Participants were recruited primarily via email listservs and were compensated in either cash or academic extra credit. All protocols were approved by the Dartmouth Committee for the Protection of Human Subjects, and all participants provided informed consent prior to participation in this study.

### Stimulus images

Stimuli for this study consisted of 24 photographs of real-world structures, including bridges, lampposts, buildings, and other similar structures. Through consultation with experts in mechanical engineering (authors SGD and VVM), a subsection of each structure was selected as the component of interest, and was outlined in red on the stimulus image. A full list of stimuli used is available on request^24^. Importantly, although the task only requires participants to consider individual items, we selected the items to intentionally cluster into meaningful categories. Based on input from the experts, the 24 stimulus items comprised three meaningful categories of structures (cantilevers, trusses, and vertical loads). Moreover, each individual item looked similar to (i.e., shared visual similarity with) at least one item from a different category. This ensured that there are two separable dimensions of similarity between the items: a dimension of visual similarity, reflecting surface-level appearance, and a separate dimension of mechanical or structural similarity, reflecting deeper conceptual knowledge about how structures support loads and maintain equilibrium. Finally, these dimensions are orthogonal to the task responses, and the categories are never mentioned to the participants.

### Expert model of mechanical similarity

To generate an expert representational model of mechanical similarity for the 24 structures from the stimulus set, we had an expert in mechanical engineering (author SGD) complete pairwise similarity ratings for all 276 pairs of stimulus images. The expert rater was explicitly asked to compare the components of interest in each pair of structures according to their mechanical and structural similarity to one another, specifically with respect to the Newtonian forces that must act on each object in order for the structure to maintain static equilibrium (i.e., so that the object remains stable and unmoving).

### High-dimensional expert model

The result of this set of expert mechanical similarity ratings was a 24-by-24 DM, which could be utilized in a standard RSA. This expert DM became our high-dimensional expert model, and was used in the computation of the searchlight RSA neural score (see below).

### Dimensionality-reduced expert model

We used multidimensional scaling (MDS) to reduce the dimensionality of the expert mechanical similarity model from 24 dimensions to 2 dimensions. This MDS projection revealed that the 24 stimulus structures clustered into three distinct categories based on their mechanical similarity features, which mapped onto existing categories of structures used by engineers: cantilevers, trusses, and vertical loads. By assigning category labels to each stimulus structure, we generated a category-level expert model that was agnostic to the discrete pairwise dissimilarity values from the original expert ratings. This category-level model enabled us to use a categorical SVM classifier to determine the degree of mechanical category information present in any DM. We used this dimensionality-reduced model to evaluate the presence of expert-level information in the informational network analysis (described below).

### FBD task

During a functional MRI scan session, participants completed a task asking them to evaluate the equilibrium state of the component of interest in each of the 24 stimulus structures. This task is referred to here as the FBD task, as it employs diagrams of Newtonian force vectors based on the FBDs used by physicists and engineers to model the forces acting on a structure. The scan session was comprised of a familiarization period with the 24 stimulus images and their components of interest, a set of practice trials involving separate stimuli and task performance feedback, and four runs of the FBD task, each containing 24 trials (one for each of the individual stimuli). During the FBD task itself, participants did not receive any feedback on their performance.

In each FBD task trial, participants were shown a stimulus image for 2 seconds without any additional markings, and then for 4 seconds with the segment of interest outlined in red. During these first 6 seconds, participants had previously been instructed to consider the Newtonian forces that must be acting on the component of interest in order for the structure to remain in equilibrium (i.e., stable and unmoving within the system. All functional imaging data used in further neuroimaging analyses were collected from this first 6 seconds of each trial. There was then a jittered fixation period, after which the component-highlighted image reappeared for 4 seconds, this time labeled either correctly or incorrectly with arrows indicating Newtonian forces (see Fig. 1 for an example labeled image). While viewing this labeled version of the image, participants evaluated whether the labeling was correct or incorrect based on the model they had imagined during the fixation period, and indicated their judgment via button press during the 4-second window. After the response period concluded, participants saw one more jittered fixation period, timed such that each trial’s total duration was exactly 15.5 seconds. Each trial was then separated by 15.5 seconds of fixation to establish a baseline for the fMRI analysis.

Each of the four fMRI task runs required participants to evaluate all 24 stimuli, where 12 images (50%) were correctly labeled on a given run. By the end of the experiment, participants had seen the correct and incorrect versions of each labeled stimulus diagram twice. Stimulus presentation order was pseudo-randomized from run to run such that each participant could not have predicted which version of a stimulus they would see at a given point in the experiment.

### Procedure

Participants completed two sessions for this experiment: a behavioral session and an fMRI scan session, each lasting ~ 1.5 h. Both sessions began with participants providing informed consent to participate, and the fMRI session additionally involved the completion of an fMRI safety screening form. After completing both sessions, participants were compensated for their time either in cash or with curricular extra credit points.

### Behavioral session

During the behavioral session, participants completed several physics and engineering concept knowledge evaluations, including two standardized, multiple-choice concept inventories: the SCI^22^ for evaluating knowledge of the mechanical properties of nonmoving systems, and the FCI^28^ for evaluating knowledge of Newtonian force properties. Not all participants completed the SCI and FCI materials (19 subjects completed the SCI and 20 subjects completed the FCI). Consequently, the models involving these tests incorporated random intercepts for concept knowledge test type to account for attrition. Additional tasks included a pairwise similarity ratings task where participants compared each unique pair of the 24 stimulus images and evaluated them for general similarity (i.e., without any specific guidelines for judging similarity).

### fMRI session

Within 1 week of the behavioral session, participants completed the fMRI scan session, which primarily involved the completion of the fMRI task described above. After completing the fMRI task, participants completed an additional set of pairwise similarity ratings for the 24 stimulus items, where this time they were instructed to evaluate similarity based on the mechanical properties of the structures in each image. Results from the similarity ratings tasks are not discussed in the present study^24^.

### fMRI image collection

A 3 T Philips Achieva Intera scanner, with a 32-channel head coil, was used to acquire brain images via gradient-echo echo-planar imaging. For functional images, an 80 × 80 reconstruction matrix was used with a 240 mm^2^ field of view to provide whole-brain coverage over 42 transverse slices (Flip angle = 90°; TE = 35 ms; TR = 2500 ms; 3 mm^3^ voxels; no gap). Slices were interleaved during acquisition. Each of the four runs of functional data collection consisted of 298 volumes. A single high-resolution T1-weighted anatomical scan was also collected for each participant (TE = 3.72 ms; TR = 8.176 ms; voxel resolution = 0.938 × 0.938 × 1.0 mm).

### Preprocessing of functional neuroimaging data

fMRI data were preprocessed using the FSL FEAT software package^29,30^. Each individual participant was preprocessed separately. First, the participant’s high-resolution T1-weighted anatomical image was skull-stripped using the FSL brain-extraction tool^31^. Then, the functional images from each of the four runs were subjected to skull-stripping, motion correction, slice timing correction, prewhitening, and highpass temporal filtering (cutoff at 100 s). Finally, the functional images were registered to the participant’s anatomical volume using the FSL linear registration tool^32–34^.

Next, beta-value estimates were calculated for each of the 24 stimulus images using an item-level univariate regression model, where an explanatory variable was set up to model the brain activity associated with each individual stimulus item. A separate regression model was computed for each functional run using the GLM. Activity was sampled from the first 6 seconds of each trial of the fMRI task, the portion of the task which participants viewed the stimulus structure and were tasked with imagining the Newtonian forces acting upon the component of interest. The jittered fixation period following this 6-second consideration period allowed for an un-confounded estimate of the BOLD signal associated with the consideration of each stimulus. The beta estimates associated with the individual stimuli were used in univariate and multivariate analyses (described below) to compute neural scores for each participant. Separate explanatory variables were also set up to model brain activity associated with the button response period (4 seconds per trial), as well as the inter-trial fixation period, combined across all trials. Brain activity associated with the button response period was excluded from further analysis. Brain activity associated with inter-trial fixation was used as a baseline for the univariate activity contrasts employed in further analyses (described below).

In the final preprocessing step, we performed cortical surface reconstruction for each participant’s T1-weighted anatomical image using the Freesurfer recon-all suite;^35^ http://surfer.nmr.mgh.harvard.edu), and transformed the resultant cortical surfaces to Surface Mapping (SUMA) format;^36^ http://afni.nimh.nih.gov/afni/suma/). Formatted cortical surface maps were fitted to standard mesh grids based on an icosahedron with 32 linear divisions, yielding 20,484 nodes for each participant’s whole-brain cortical surface.

### Neural score methods

Three different varieties of neural scores were computed and used to predict performance on the FBD task. The primary score of interest in this paper was the informational network score, derived from a novel representational analyses referred to here as an informational network analysis. This score was validated by comparison to two additional scores: a univariate score and a multivariate RSA score. Below is a full description of the steps involved in the computation of each score, beginning with the simplest method (the univariate score) and proceeding in order of complexity (concluding with the informational network score).

### Univariate neural score

To calculate the univariate neural score for each individual participant (Fig. 3), we proceeded according to the following steps:

1. For each of the four fMRI runs, a whole-brain univariate contrast was computed for the contrast of stimulus images > between-trial fixation baseline, comparing the beta-value estimates for each contrast condition from the univariate GLM described above.
2. A “robust range” threshold was applied to the contrast results for each run using the fslstats function from the FSL toolbox to identify the top 2% of voxels with the greatest positive *z* value for the contrast.
3. The *z* values for all voxels in the robust range for each run were averaged, resulting in a run-level univariate neural score for each of the four runs.
4. Each participant’s univariate neural score from the first fMRI run was used in further analyses.

### Searchlight RSA neural score

To calculate searchlight RSA neural score for each individual participant (Fig. 3), we proceeded according to the following steps:

1. For each of the four fMRI runs, a whole-brain searchlight analysis was performed with searchlight sphere (radius = 100 voxels) implemented in Python using the PyMVPA toolbox^37,38^. At each searchlight location, a DM was computed for the 24 stimulus items by calculating the 276 pairwise correlation distances between the beta values associated with each stimulus item.
2. A RSA compared the DMs at each searchlight location with the mechanical similarity expert model using a Spearman correlation, resulting in a whole-brain RSA correlation map for each fMRI run.
3. The whole-brain RSA correlation map from each run was passed through a Fisher z-transformation and a threshold of *z* ≥ 2 was applied.
4. The *z* values for all searchlight locations in the *z* *≥* 2 mask were averaged for each run, resulting in a run-level RSA neural score for each of the four runs. Participants with no searchlight locations above the z ≥ 2 threshold were dropped from all analyses; two participants (one from each group) were dropped for this reason.
5. Each participant’s RSA neural score from the first fMRI run was used in further analyses.

### Informational network neural score

To calculate informational network neural score for each individual participant (Fig. 2), we performed a novel multivariate representational analysis that we have termed an informational network analysis. The analysis proceeded according to the following steps:

1. We began with the univariate robust range results for the images > fixation contrast for each fMRI run, as derived in steps 1–2 of the univariate neural score calculation. Binary masks were created for each run containing only the voxels in the robust range.
2. Using the whole-brain searchlight DMs for each run (described in step 1 of the RSA neural score calculation), we selected only those DMs from searchlight locations with centers inside the univariate robust range mask for that run.
3. The surviving DMs were subjected to a bootstrapped hierarchical clustering algorithm using the R package pvclust^23^, which assigned each DM to an informational network based on inherent representational structure. This bootstrapped method determines the number of informational networks uniquely for each set of DMs based on bottom–up statistical reliability. Informational network assignment was performed separately and independently for each run, and did not involve any a priori model of representational similarity.
4. An average DM was calculated for each informational network from each run.
5. Each average DM was projected into two dimensions using MDS, implemented in R using the MASS package^39^. MDS was employed as a noise-reduction step to account for assumed redundancies in the high-dimensional feature space. In previous analyses with this data set, we found that two feature dimensions were sufficient to show meaningful differences in category-level information between informational networks^24^.
6. The MDS projection of each DM was then evaluated for its expert-level representational structure using a radial SVM (SVM) classifier^40,41^, which predicted mechanical category labels for each item in a given DM. The SVM classification accuracy value for the most accurate network DM from each fMRI run was designated as the run-level neural score.
7. Each participant’s informational network neural score from the first fMRI run was used in further analyses.

The univariate mask utilized in the univariate neural score and informational network neural score was used as an agnostic, computationally simple method of reducing data to include only task-related signal, unbiased as to stimulus category membership. To confirm that this approach did not miss any informative neural activity patterns, we compared the results from this selection procedure with those produced using a whole-brain RSA analysis. The univariate mask proved the most effective (comparisons are reported in Supplementary Table 1). In addition, the searchlight radius of 100 voxels used in both the RSA and informational network neural scores is a common size, and other studies have found patterns to be robust across variable searchlight sizes (including those of similar volume to ours)^42^. Finally, the MDS and clustering components of the informational network score computation are intended to reduce the dimensionality of the data by collapsing across less informative dimensions. We followed a clustering method used previously in our lab^4,43^.

### Linear regression models for neural scores predicting concept knowledge

We estimated each neural score’s ability to predict concept knowledge using linear mixed-effect modeling in R. Separate models were built for each neural score, where neural score was included as a fixed effect with concept knowledge test score accuracy as the dependent variable. Random intercepts were included for each participant and for each type of concept knowledge test (i.e., FBD task, SCI, and FCI). This composite-dependent variable was made possible by the fact that all three concept knowledge measures shared the same scale (an accuracy score between 0 and 1). As discussed, all neural scores and all FBD task accuracy scores were drawn from the first fMRI run only.

### Neural score localization

For each neural score, results for all participants were projected onto a common cortical surface (a surface map of the MNI brain) in order to identify which brain regions contributed to participants’ neural scores. Each surface node contributing to any participant’s neural score was then color-coded to reflect the group of the participant or participants from which it was generated. If a location was observed only in engineering students, its surface node was colored red. If a location was observed only in novices, its location was colored light blue. Locations observed in participants from both groups were colored purple.

## Supplementary information

Supplementary Information

## Data Availability

The data sets and code generated during the current study are available from the corresponding author on reasonable request.
