# Supplementary material for: Decoding individual differences in STEM learning from functional MRI data
Source: Nat Commun. 2019 May 2;10:2027. doi: 10.1038/s41467-019-10053-y (PMC6497647; doi:10.1038/s41467-019-10053-y)
Supplement: Supplementary file 1 — Supplementary Information [file 41467_2019_10053_MOESM1_ESM.pdf]

Supplement to: Decoding individual differences in STEM learning from functional MRI data

Cetron et al.

Supplementary Information

In this supplement, we present one supplementary table and three supplementary figures that provide additional clarity and context for results and methods discussed in the main text. Specifically, the following information is included: Supplementary Table 1 shows the regression results for two alternative neural score computations not discussed in the main analyses. Supplementary Figure 1 shows enlarged example stimuli from the free body diagram (FBD) task referenced in the inset of Figure 1 in the main text. Supplementary Figure 2 shows the differences in FBD task performance by group across each fMRI run, as reported in the Results section of the main text. Finally, Supplementary Figure 3 shows histograms of the brain regions contributing to each neural score discussed in the main text. Data and further information are available upon reasonable request (email corresponding author, David J. M. Kraemer: David.J.M.Kraemer@Dartmouth.edu).

Supplementary Table 1

Regression Results for Alternative Neural Scores (not included in paper)

Model	Voxel Selection Method	Score Derivation Method	β Parameter Estimate	Effect Size ($\beta_{std.}$)	p
Informational Network Score using RSA Mask	RSA results from comparison to expert model: $z \geq 2$	Informational network analysis	.81	.21	.2
RSA Score using Univariate Mask	Univariate contrast (images > baseline): top 2% of voxels	Average RSA z-value	-.011	-.04	.8

Note: In addition to the three neural score methods described in Tables 1 and 2, we computed two other neural scores in order to discern the effect of voxel selection on each multivariate score derivation method. Specifically, we computed the following two scores: 1) a neural score using the informational network analysis on the voxels in the searchlight RSA mask ($z \geq 2$), and 2) a neural score consisting of the average RSA z-value for the univariate images > baseline contrast (top 2% of voxels). Neither of these methods yielded scores that effectively predicted performance on the concept knowledge task. This result suggests that the effectiveness of a neural score depends on the conjunction of both voxel selection and score derivation methods.

Supplementary Figure 1: Example stimuli from the FBD task

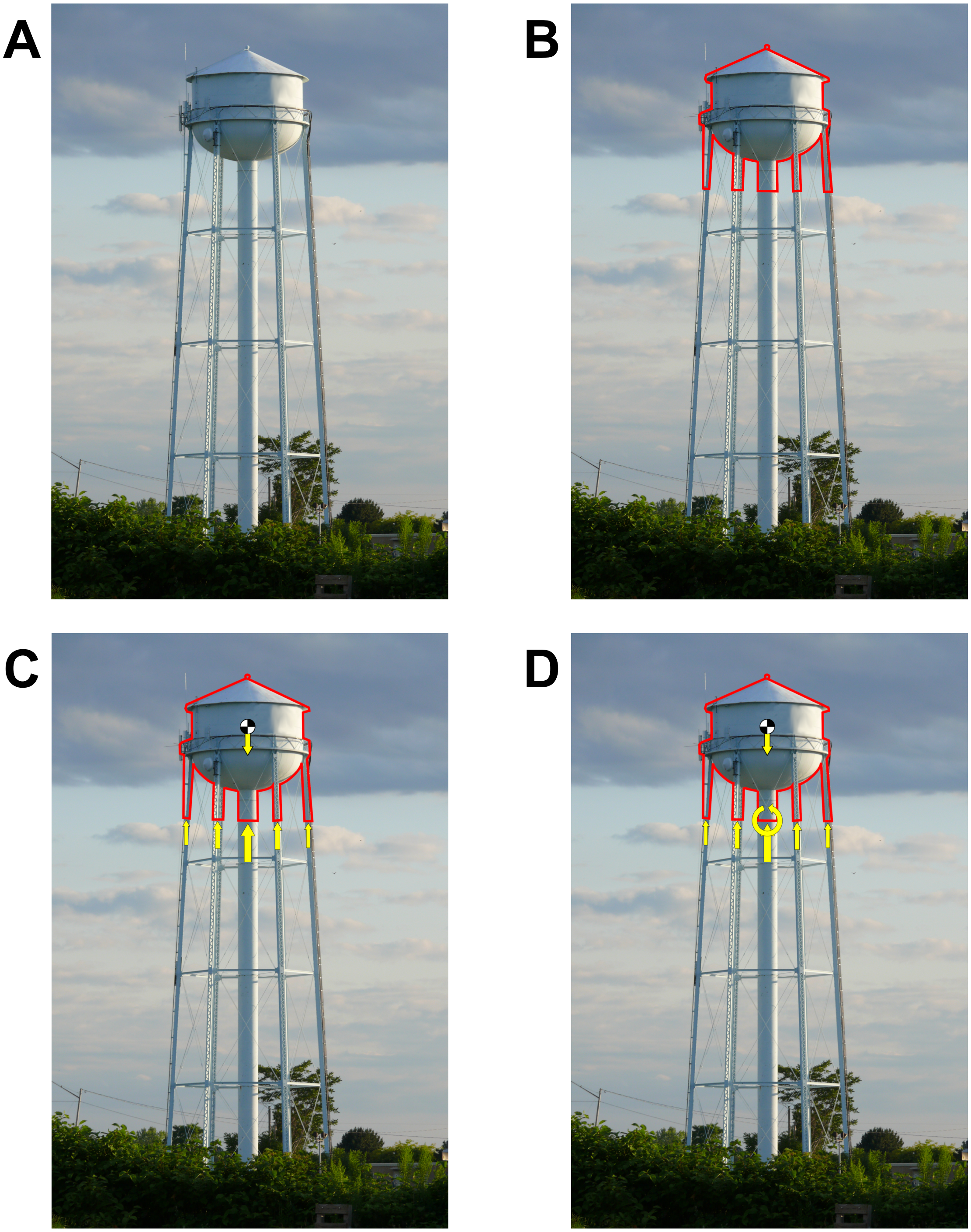

Note: Larger example of the type of stimuli used in the FBD task participants completed during fMRI scanning to complement the example provided in the inset of Figure 1 in the main text. Participants first saw images A and B, during which time they were instructed to consider the

forces that would be acting on the red highlighted portion of the structure to keep the system in equilibrium. All neural activity data used to compute neural scores was drawn from this consideration period. Then, after a jittered fixation, yellow arrows appeared overlaid on the image, either labeling the forces acting on the image correctly (C) or incorrectly (D). Participants had to indicate via button press whether the image they were viewing was correctly or incorrectly labeled.

Supplementary Figure 2: Free body diagram (FBD) task accuracy at each fMRI run by group

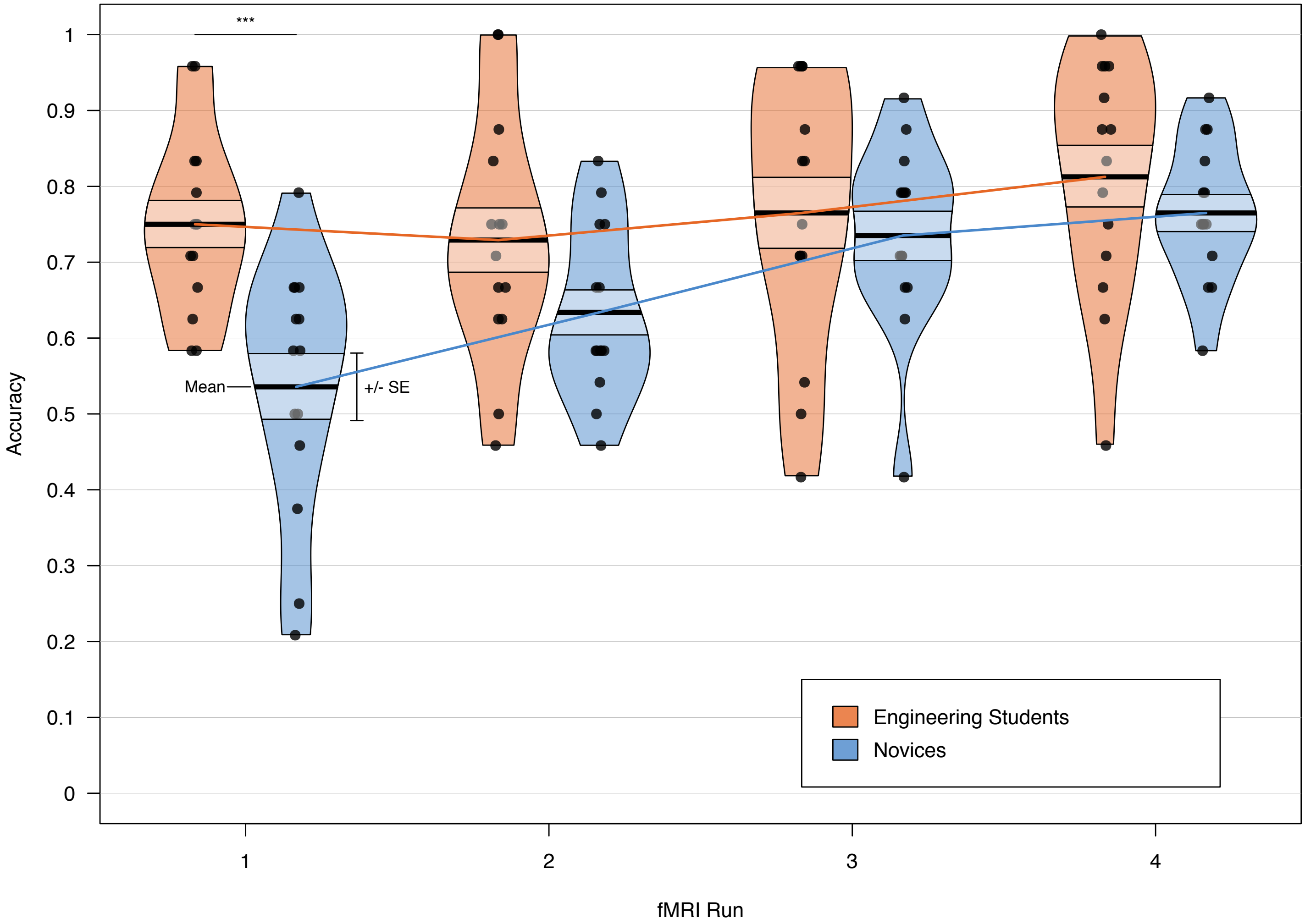

Note: Performance on the FBD task by group at each fMRI task run. On average, engineering students outperformed novices on the FBD task ($M_{Eng.}$ = 76.4%, $M_{Nov.}$ = 66.7%, $t(24.48) = 2.11$, $p = .045$). But group differences were maximal at Run 1 ($M_{Eng.}$ = 75.0%, $M_{Nov.}$ = 53.6%, $t(23.53) = 3.92$, $p = .0007$). Both groups improved on the FBD task despite never receiving feedback on their performance, but novices improved the most, and resultantly group differences in FBD task performance disappeared in runs 2-4 (Run 2: $M_{Eng.}$ = 72.9%, $M_{Nov.}$ = 63.4%, $t(23.24) = 1.82$, $p = .08$; Run 3: $M_{Eng.}$ = 76.5%, $M_{Nov.}$ = 73.5%, $t(23.06) = 0.51$, $p = .6$; Run 4: $M_{Eng.}$ = 81.3%, $M_{Nov.}$ = 76.5%, $t(21.08) = 0.99$, $p = .3$).

Supplementary Figure 3: Histograms of brain regions contributing to each neural score method

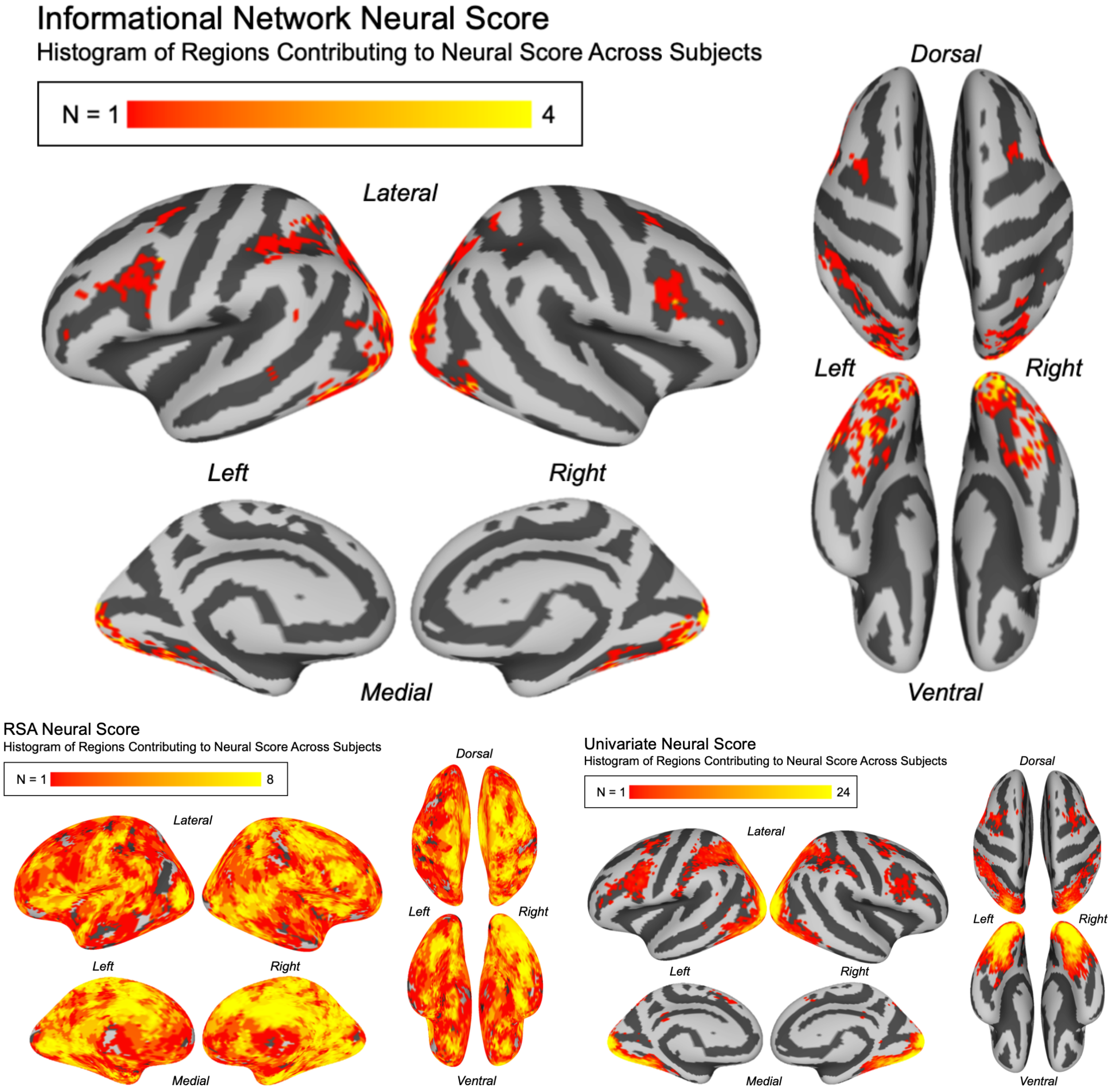

Note: All of the neural scoring methods we derived were designed specifically to be data-driven and computed at the individual level. As a result, each neural score computation was based on a set of brain regions unique to a particular individual's brain activity during a particular fMRI run. In order to visualize which regions were involved in these computations generally across all participants, the brain regions contributing to each participant's neural scores have been overlaid on a cortical surface map of the MNI brain to form a histogram for each neural scoring method. The color value of a particular brain region represents the number of participants for whom that

brain region contributed to their neural score computation. Note that all neural score results shown here come from the first fMRI run. The informational network analysis (*top*) showed the most idiosyncratic distribution of neural response patterns, the RSA method (*bottom left*) displayed the most breadth of coverage, and the univariate method (*bottom right*) showed the most between-subject convergence.
